# Natural Ergot Alkaloids in Ocular Pharmacotherapy: Known Molecules for Novel Nanoparticle-Based Delivery Systems

**DOI:** 10.3390/biom10070980

**Published:** 2020-06-30

**Authors:** Iara Baldim, Wanderley P. Oliveira, Varsha Kadian, Rekha Rao, Nitesh Yadav, Sheefali Mahant, Massimo Lucarini, Alessandra Durazzo, Raquel Da Ana, Raffaele Capasso, Selma B. Souto, Antonello Santini, Eliana B. Souto

**Affiliations:** 1 Faculty of Pharmaceutical Sciences of Ribeirão Preto, University of São Paulo, Av. do Café s/n, Ribeirão Preto, SP 14040-903, Brazil; iara.baldim@usp.br (I.B.); wpoliv@fcfrp.usp.br (W.P.O.); 2 CEB—Centre of Biological Engineering, University of Minho, Campus de Gualtar, 4710-057 Braga, Portugal; 3 Department of Pharmaceutical Sciences, Guru Jambheshwar University of Science and Technology, Hisar 125001, Haryana, India; kadyanvarsha313@gmail.com (V.K.); rekhaline@gmail.com (R.R.); ny02111997@gmail.com (N.Y.); 4 Department of Pharmaceutical Sciences, Maharshi Dayanand University, Rohtak 124001, Haryana, India; sheefali.m@gmail.com; 5 CREA-Research Centre for Food and Nutrition, Via Ardeatina 546, 00178 Rome, Italy; massimo.lucarini@crea.gov.it (M.L.); alessandra.durazzo@crea.gov.it (A.D.); 6 Department of Pharmaceutical Technology, Faculty of Pharmacy, University of Coimbra, Pólo das Ciências da Saúde, Azinhaga de Santa Comba, 3000-548 Coimbra, Portugal; quele.ana@gmail.com; 7 Department of Agricultural Sciences, University of Napoli Federico II, Via Università 100, 80055 Portici, Italy; 8 Department of Endocrinology, Hospital de São João, Alameda Prof. Hernâni Monteiro, 4200–319 Porto, Portugal; sbsouto.md@gmail.com; 9 Department of Pharmacy, University of Napoli Federico II, Via D. Montesano 49, 80131 Napoli, Italy

**Keywords:** ergot alkaloids, ocular pharmacology, glaucoma, lipid nanoparticles, polymeric nanoparticles

## Abstract

Several pharmacological properties are attributed to ergot alkaloids as a result of their antibacterial, antiproliferative, and antioxidant effects. Although known for their biomedical applications (e.g., for the treatment of glaucoma), most ergot alkaloids exhibit high toxicological risk and may even be lethal to humans and animals. Their pharmacological profile results from the structural similarity between lysergic acid-derived compounds and noradrenalin, dopamine, and serotonin neurotransmitters. To reduce their toxicological risk, while increasing their bioavailability, improved delivery systems were proposed. This review discusses the safety aspects of using ergot alkaloids in ocular pharmacology and proposes the development of lipid and polymeric nanoparticles for the topical administration of these drugs to enhance their therapeutic efficacy for the treatment of glaucoma.

## 1. Introduction

Ergot alkaloids are a large group of compounds, comprising more than 40 highly biologically active molecules [1,2,3], produced by microfungi belonging to the genus *Claviceps* and relative species [4]. Chemically, these molecules share a four-membered ring—ergoline—known to interact with neurotransmitter receptors. These natural compounds can interact with serotonergic, dopaminergic, and adrenergic receptors as agonists or antagonists. Their pharmacological profile is attributed to the similar structure between lysergic acid-derived compounds and these neurotransmitters [4].

Ergot alkaloids are mycotoxins of high agro-economic interest, which can be present in food and feed, compromising the health of consumers, both humans and animals [5,6,7,8,9,10,11,12]. Several properties, such as antibacterial, antiproliferative, and antioxidant activities, are attributed to alkaloids [13,14]. Among the toxic effects of ergot alkaloids, nausea, vomiting, digestive disorders, weight loss, muscle pain and weakness, numbness, itching, and rapid or slow heartbeat were reported [15,16].

The toxicological profile of ergot alkaloids was the subject of investigation. The ability of ergot alkaloids to cross the blood–brain barrier (BBB) was studied in vitro by Mulac et al. using primary porcine brain endothelial cells [17]. The authors identified the active transport of ergometrine as a substrate for the breast cancer resistance protein (*BCRP*)/ATP-binding cassette subfamily G member 2 (*ABCG2*) transporter, demonstrating that ergot alkaloids can cross the BBB in high quantities in only a few hours. The 8-(*S*) isomers of ergot alkaloids were found to interfere with the BBB integrity, demanding the risk assessment of ergot alkaloids in food and feed. The authors found that ergocristinine can potentially accumulate in brain endothelial cells. Earlier, a study conducted also by Mulac et al. described the in vivo toxic effects of the six most predominant ergot alkaloids, namely, ergotamine, ergocornine, ergocryptine, ergocristine, ergosine, and ergometrine, together with their -inine isomeric forms [18]. The authors evaluated the in vitro cytotoxicity profile of these six alkaloids in the renal proximal tubule epithelial cells and in normal human astrocytes for comparison with the in vivo data. While ergometrine as a lysergic acid amide did not show any effect, the peptide ergot alkaloids revealed a different toxic potential. Among all tested alkaloids, ergocristine presented the highest cytotoxicity, inducing apoptosis in human kidney cells starting at a concentration of 1 μM in the renal proximal tubule epithelial cells. The study highlights the effects of ergot alkaloids regarding cytotoxicity and accumulation in human primary cells. In addition to the well-described receptor effects, the results also identified apoptosis, which points to the complex mode of action of ergot alkaloids.

Despite the recognized toxic risk for human and animal health, ergot alkaloids are efficiently used in pharmaceutics, in particular against glaucoma, a serious optic neuropathy with associated high intraocular pressure (IOP) as the main risk factor [19,20]. The high IOP injures the optic nerve, causing long-term damage and disruption of communication between the retina and the brain, which can result in irreversible vision loss [21]. Furthermore, glaucoma is recognized as the second cause of blindness, in developed countries, and its management requires lowering IOP by enhancing the drainage of fluid from the eye or decreasing the generation of the fluid. Different classes of drugs can be used to lower IOP: β-blockers, adrenergic agonists, carbonic anhydrase inhibitors, prostaglandin analogues, miotics, and hyperosmotic drugs [22,23,24]. Each of these categories of drugs presents inherent adverse effects, which leads to the search for more effective and safer alternatives. In this context, scientific research is focused on herbal bio-actives, such as ergot derivatives, since these molecules are suggested as a potent group of anti-glaucoma drugs, with a safety profile.

Therapeutically important alkaloids include ergonovine (ergometrine), ergotamine, and ergocristine [3]. Their synthetic derivatives, namely, methylergonovine, methysergide, dihydroergotamine, bromocriptine, and lysergic acid diethylamide, are also used in therapy [4]. The first evidence of the therapeutic use of ergot alkaloids dates back to the early 20th century (1926) when ergotamine tartrate was tested for its potential to reduce IOP [25]. Later, dihydroergocristine, followed by a mixture consisting of equal parts of three hydrogenated alkaloids of dimethyl pyruvic acid series, was investigated [26]. Several other natural compounds were proposed to reduce IOP, with ergot alkaloids being the most effective. In this review, we discuss the preclinical effects of several natural ergot alkaloids as therapeutic agents in ocular pharmacology and anticipate the advantages of their encapsulation in lipid and polymeric nanoparticles to improve their topical bioavailability while limiting the well-known toxicological effects.

## 2. Role of Ergot Alkaloids in Reducing Intraocular Pressure

**Figure 1:**
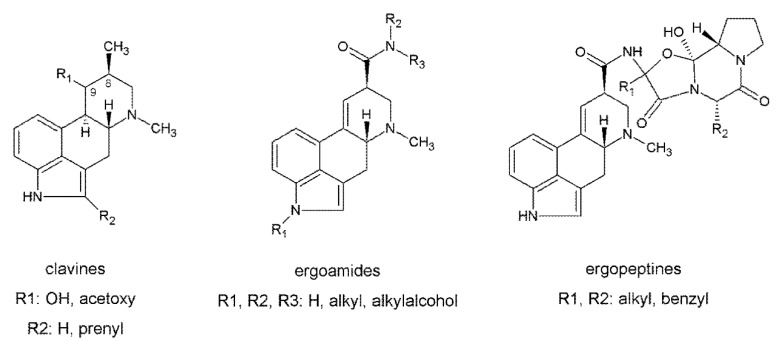
Chemical structure of the different classes of ergot alkaloids. Modified after Gerhards et al. [36].

Glaucoma is characterized by progressive degeneration of the optic nerve head and the retinal nerve fiber associated with the loss of vision [24]. An important risk factor associated with this disease is the high IOP, which is why therapy is based on IOP reduction. All ergot alkaloids have in common an indole-derived tetracyclic ring structure (ergoline) and, according to their structural features, the naturally occurring ergots are categorized into three main classes: amide- and peptide-like amide derivatives of d-lysergic acid, and the clavine alkaloids [27] (Figure 1). The pharmacological profile of ergot alkaloids is linked to the structural similarity between d-lysergic acid-derived compounds and neurotransmitters like noradrenaline, dopamine, and serotonin [28]. This structural similarity anticipates that these neurotransmitters can interact as agonists or antagonists in these receptors, depending on the substituent attached to the carboxyl group of d-lysergic acid [29].

The first evidence of the intraocular-lowering effect of ergot alkaloids was observed with Hydergine^®^, both in rabbits and in humans [30]. This is a sympatholytic drug composed of a combination of equal parts of three dehydrogenated derivatives of ergot alkaloids: dihydroergocristine, dihydroergocornine, and dihydroergocryptine methane sulfonates. Moreover, ergoline derivatives with a predominant dopaminergic activity, such as bromocriptine, lergolide, pergolide, cianergolide, and lisuride, were shown to decrease IOP in rabbits, monkeys, and humans [31,32,33,34]. A United States (US) patent also reported the production of a formulation comprising bromocriptine as the active ingredient, suitable for ocular instillation and used as an anti-glaucomic agent [35].

Bromocriptine, which is marketed as its mesylate salt form, is known for its dopaminergic action [37,38] and its ability to decrease IOP upon local administration. This effect was firstly described in rabbit eyes and further supported by the evidence of its efficacy to reduce IOP in humans upon oral administration. However, the unavailability of a suitable dosage form hampers the therapeutic use of bromocriptine. Since oral administration of this drug is associated with undesirable side effects, a topical delivery system would be more appropriate. Bromocriptine poses significant challenges owing to its insolubility in the aqueous media conventionally utilized for ocular administration.

Given this, Puras et al. investigated the effect of natural ergot alkaloids on IOP and on aqueous humor dynamics in the rabbit model [39]. In the study, ergocristine, α-ergocriptine, and ergocornine were found to be effective in decreasing the IOP in a dose-dependent fashion, producing an ocular antihypertensive effect. The same group later conducted a comparative evaluation of the effect of these alkaloids on IOP and on aqueous humor dynamics, both in ocular normotensive and in α-chymotrypsin-induced ocular hypertensive rabbits [40]. The tested molecules decreased both tonographic outflow facility and aqueous humor inflow, which explains the reduction of the IOP in a dose-related fashion for both rabbit models. The use of natural ergot alkaloids as therapeutic agents was reported to require narrowing the specificity of the compounds by chemical modifications, in order to retain their therapeutic properties while avoiding adverse side effects [41].

Santafé et al. studied the effects of dihydroergocristine, timolol, and pilocarpine on the IOP and on the pupil diameter in conscious rabbits [42]. The authors showed that the ergo derivative dihydroergocristine reduced the IOP more significantly than timolol, and much more than pilocarpine. The ocular hypotensive effect of dihydroergocristine was accomplished by a great reduction in aqueous humor formation, while timolol mainly reduced aqueous humor formation and pilocarpine increased aqueous humor outflow. Dihydroergocristine did not change the pupil diameter, whereas timolol induced slight mydriasis while pilocarpine provoked myosis. The authors proposed that dihydroergocristine might block the α-adrenoceptors in the ciliary body, although available data about pre-treatment with either metoclopramide or domperidone suggest the participation of DA2 dopamine receptors in the ocular hypotensive effect of ergot derivatives.

Rowell and Larson studied the effect of several ergot alkaloids on the dopaminergic activity [43]. The authors demonstrated that ergocryptine, ergocristine, and bromocriptine induced an elevation in baseline dopamine with an effective concentration of about 30 µM, whereas ergotamine, ergonovine, ergovaline, and ergocornine did not show any activity. Ergocryptine affects dopaminergic activity mainly through interaction with D_2_-type receptors. The authors showed that the time-course of the ergocryptine-stimulated release was relatively slow compared to amphetamine, nicotine, or K^+^-stimulated [^3^H] dopamine release. Many receptor antagonists were examined for their ability to block ergocryptine-stimulated release. The results indicate that various ergot alkaloids can not only interact with dopaminergic receptors, but also produce dopaminergic effects by increasing the release of dopamine from central nerve endings. The authors also discussed several mechanisms to account for the evoked neurotransmitter release.

## 3. Encapsulation of Ergot Alkaloids for Ocular Administration

There are many scientific reports confirming the anti-glaucoma action of ergot alkaloids; this property, however, can still be further improved by the encapsulation of these drugs in a suitable delivery system able to improve the drug’s bioavailability and targeted delivery while reducing the side-effects. Conventional glaucoma therapy involves the administration of topical eye-drops, which leads to pre-corneal loss of the drug, accounting for its poor bioavailability and reduced therapeutic effect. Only 1–5% of the topically administered drug can penetrate the cornea and reach the internal ocular tissues [44]. This low bioavailability is due to distinct barriers for drug penetration into the eye. As a result, the pre-corneal drug half-life is approximately one minute [45]. The rapid clearance requires frequent administration of eye drops to sustain the reduction in IOP, which can trigger sensitivity reactions, mechanical injury through the misuse of the eye drops, and lack of patient compliance [44,45]. In addition to the lower systemic bioavailability, the currently available marketed formulations with anti-glaucoma drugs show limited capacity in crossing the blood–retinal barrier. The anterior sphere is the smallest of the two and is bordered anteriorly by the cornea, whereas the larger posterior sphere is an opaque fibrous shell encased by the sclera. When considering ocular drug delivery, there is a substantial number of issues related to the precorneal area of the eye that can have major impact on the drug’s bioavailability in the expected target issues. Since both lacrimation and blinking profoundly influence the residence time of liquid ocular drug delivery systems, their comfort on the eye seems essential [46,47]. Placing the drug delivery system as deeply as possible into the lower cul-de-sac will assist in patient comfort, as well as increase the residence time. However, liquid delivery systems do not adhere to the cornea and to the conjunctival surface for sufficient time in order to have an effective systemic absorption.

**Figure 2:**
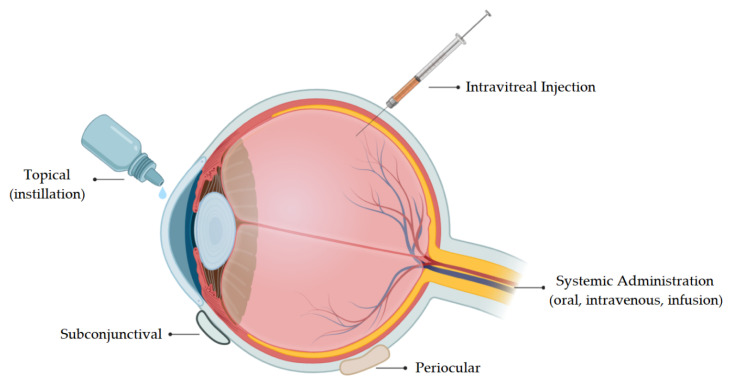
Schematic representation of the different administration routes for drug delivery to the eye.

The eye is considered an immune privileged organ mainly due to the blood–retinal barriers, the avascular character of the cornea, the absence of a lymphatic drainage from the anterior chamber, the presence of soluble immunomodulatory factors in aqueous humor (released from the cells and tissues surrounding the anterior chamber and secreted by the ciliary body), and the cell-surface immunomodulatory factors expressed on parenchymal cells [48]. Among these, blood–ocular barriers play a protective role that prevents the entry of molecules from the systemic circulation into the ocular compartments. These barriers are classified into the blood–aqueous barrier (BAB) and the blood–retina barrier (BRB), located in the anterior and posterior segments of the eye, respectively. The treatment of diseases involving structures of the posterior segment of the eye (such as choroid, vitreous humor, and retina) is one of the main bottlenecks in pharmaceutical technology [49]. Figure 2 illustrates the different administration routes for drug delivery to the eye.

Drug delivery to the posterior segment remains a challenge due to the nature of the blood–ocular barrier, requiring improved approaches to overcome the many barriers for the delivery of therapeutic drug concentrations to intraocular tissues. The success of the pharmacological therapy of diseases of the posterior eye segment depends on the efficiency of the drug to reach the intended site of action, i.e., target tissue, contact time, and disease itself [50]. To overcome the limitations encountered with classical drug delivery systems, novel ocular delivery systems, such as lipid and polymeric nanoparticles, are proposed to enhance the therapeutic efficacy of the anti-glaucoma drugs.

**Figure 3:**
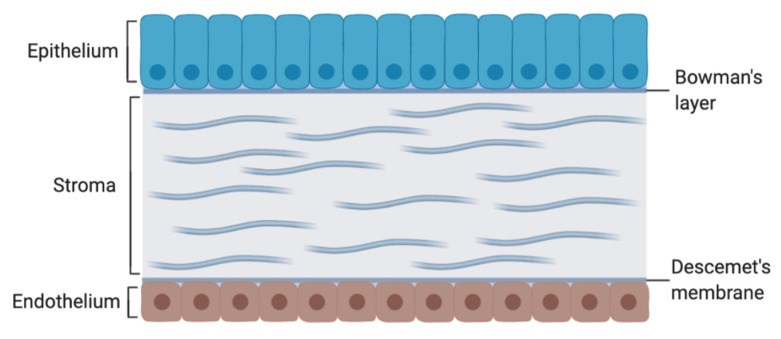
Anatomy of the cornea.

The corneal epithelium provides maximum resistance to drug penetration. As shown in Figure 3, it comprises tightly adherent cells, through which the drug molecules can penetrate via two ways: either partitioning through the cells (intracellular), with predominance for lipophilic drugs, or bypassing between the cells (paracellular), predominantly for hydrophilic and/or small-molecular-weight drugs. Moreover, the lipophilic nature of the epithelial cells can block the passage of about 90% of hydrophilic drugs and about 10% of lipophilic drugs [51]. The stroma is an aqueous environment that limits the diffusion rate of highly lipophilic molecules. Bowman’s layer and Descemet’s membrane do not provide resistance for drug penetration, and the endothelium may play a small role in rate-limiting lipophilic compounds [51].

### 3.1. Lipid Nanoparticles

**Figure 4:**
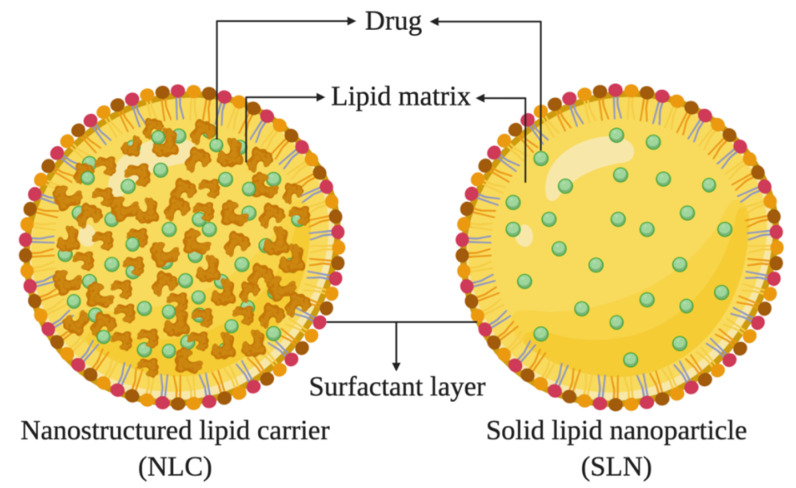
Schematic representation of the different morphology of lipid nanoparticles (upper: nanostructured lipid carriers (NLCs) and solid lipid nanoparticles (SLNs)).

Nanosized particles were explored for the entrapment of drugs as a novel strategy to increase their targetability, bioavailability, and therapeutic effect, as well as the range of molecules to be clinically used [52]. Since their introduction as drug delivery systems, solid lipid nanoparticles (SLNs) and nanostructured lipid carriers (NLCs) were explored for use in the most diverse administration routes (Figure 4). Among them, these lipid systems represent an interesting approach for the ocular route, due to their ability to improve the corneal penetration of drugs [48,52,53,54,55,56]. SLNs and NLCs are delivery systems that combine the advantages of liposomes and emulsions (biocompatibility and possibility to scale-up) with the advantages of polymeric nanoparticles (protection of the drug and modulation of the release profile) [57]. These versatile lipid carriers exhibit improved drug loading and permeation characteristics, followed by acceptable safety profile [58,59,60,61], which are reasons that allow their use for ocular delivery. Moreover, the possibility to be produced under sterilized conditions and/or being sterilized by autoclaving [62] further enhances their interest for ocular administration of drug [63]. The mucoadhesive properties of lipid nanoparticles are an additional advantage to improve their intimate contact with the ocular mucosa. The prolonging of the corneal contact time of the loaded drug can further be enhanced by developing cationic nanoparticles [64,65,66,67], increasing the bioavailability and reducing undesirable effects [68,69,70,71,72].

By definition, SLNs are composed of a solid lipid, with a melting point above 40 °C, dispersed in an aqueous surfactant solution, and falling within the nanometric size range [72,73]. NLCs are based on a blend of solid and liquid lipids composing a lipid matrix which also melts above 40 °C. Morphologically, SLNs are characterized by imperfections in the lipid crystal, which contributes to increasing the loading capacity, mainly for lipophilic drugs. The solubility of the drug in the lipid materials are the governing factor for their selection, with the purpose of increasing the loading capacity and encapsulation efficiency of drugs into SLNs/NLCs, improving the drug bioavailability and reducing adverse side effects as less concentration is needed to exhibit the desired therapeutic effect [74,75].

The recent scientific literature substantiates the suitability of these nanoparticles for the ocular delivery of different drugs. To understand the distinctive features of SLNs/NLCs, Table 1 provides a summary of different classes of drugs loaded in lipid nanoparticles for the treatment of ocular disorders. Although there is a consistent lack of studies in the literature concerning the encapsulation of ergot alkaloids in lipid nanoparticles, an overview in the available literature in lipid nanoparticles could anticipate their benefits as an improved and safer alternative for glaucoma therapy via ocular route.

Taking into account the anatomical and morphological characteristics of the corneal barrier (Figure 2), some properties of the colloidal systems are important parameters to be considered to the development of formulations for ocular delivery. The human eye can tolerate particles of about 10 µm; however, particles containing an average diameter between 50 and 400 nm are more suitable for ocular instillation. The sub-micrometer size allows mucoadhesion and crossing of the eye barriers, enabling efficient drug delivery to the target site [72,76,77]. Furthermore, the appropriate average size and the polydispersion of the particles avoid corneal irritation [78]. Furthermore, positively charged nanoparticles present better penetration through the cornea due to the electrostatic interaction established with mucin, a negatively charged protein present in the epithelial cells of the cornea. These interactions increase the pre-corneal residence time of the ophthalmic formulation and offer enhanced drug penetration and absorption [54,67]. Table 2 depicts the composition, mean particle size, polydispersity index, and zeta potential of examples of cationic lipid nanoparticles loading ophthalmic drugs.

Cationic lipid nanoparticles offer the potential to enhance biocompatibility of drugs when instilled topically for ophthalmic purposes. Introduction of positive charge on the surface of nanoparticles promotes electrostatic interactions between the anionic ocular mucosa and cationic nanoparticles, thereby reducing the contact angle with cornea. As a result, a considerable elongation in residence time and uptake of drug moieties is observed [72]. Therefore, cationic lipid nanoparticles may prove highly promising vehicles for ophthalmic delivery of drug, particularly for poorly water-soluble moieties [88]. Table 2 lists recent studies reporting the development of cationic lipid nanoparticle for ocular delivery.

The selection of ophthalmically acceptable excipients plays a vital role in successful design of functional and stable lipid nanoparticles. There is currently increased interest in promoting a positive charge onto lipid nanoparticles by coating them with a cationic moiety, e.g., chitosan [89], l-arginine [90], and/or other cationic lipids (cetyl trimethyl ammonium bromide and stearylamine) [91]. Stearylamine is a lipid with surface-modifying property that is commonly used to produce positively charged lipid nanoparticles. Studies reported stearylamine as a well-tolerated and safe cationic lipid after repeated topical ocular administration in rabbits [92]. Cationic materials are able to enhance bioadhesion of lipid nanoparticles to corneal tissues, resulting in their prolonged retention in the eyes.

Biodegradability is another characteristic feature required for ophthalmic nanoparticles to limit their accumulation in the eyes, especially in chronic eye disorders [93]. Octadecyl quaternized carboxymethyl chitosan is a cationic material possessing appreciable biodegradability and biocompatibility, and it is devoid of toxicity. This cationic material was reported suitable for prolonging drug effectiveness, minimizing drug associated side effects, improving drug absorption, and enhancing bioavailability [94,95,96].

The major concern in using cationic lipid nanoparticles is the toxicity associated with surfactants and other cationic molecules [64,65]. It is well documented that non-ionic surfactants are comparatively less toxic than the ionic ones [97,98]. However, cationic surfactants produce higher toxicity. Among cationic surfactants, CTAB (cetyl trimethyl ammonium bromide) and DDAB (dimethyl dioctadecyl ammonium bromide) are commonly reported for fabrication of cationic lipid nanoparticles for various applications [99]. Silva et al. evidenced CTAB as highly toxic in comparison with DDAB, used at the same concentration [100].

Surfactants are employed to disperse the lipid matrix in water via reduction of surface tension and energy, preventing aggregation of lipid nanoparticles [101]. Surfactants are amphiphilic molecules comprising hydrophilic and hydrophobic portions in their composition. On the basis of the presence and absence of charged groups present in the head region, these molecules are characterized as being ionic and non-ionic, respectively [101,102]. Ionic surfactants are further categorized as (i) anionic (negatively charged), (ii) cationic (positively charged), or (iii) amphoteric (possess both negative and positive charge), depending on the charge in their head group [103]. Among these, the property of amphoteric surfactants is pH-based and shows a positive charge with low pH and negative charge with high pH, with no charge at intermediate pH. This information is particularly important for selection of surfactants used in the design and fabrication of lipid nanoparticles [100].

Since surface characteristics of lipid nanoparticles have decisive role in their interaction with biological membranes, the type and concentration of surfactants should be taken into account during their selection. Depending on the surface charge, a molecular structure has a certain degree of lipophilicity. As a result, different surfactants may interact variably with cell membranes, displaying variable degrees of cytotoxicity. Moreover, surface properties of lipid nanoparticles also play a key role in their in vitro and in vivo performance [100].

### 3.2. Polymeric Nanoparticles

**Figure 5:**
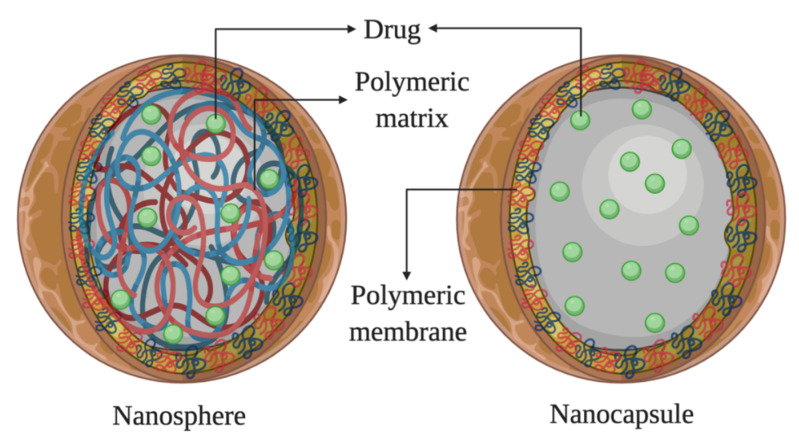
Schematic representation of the distinct morphology of a nanosphere and a nanocapsule.

Polymeric nanoparticles currently represent one of the most widely used strategies to enhance drug absorption through biological membranes [109]. Other benefits of such systems include increased bioavailability, excellent mechanical stability, and high drug payload [110]. Furthermore, the ability of these nanoparticles to improve ocular bioavailability of a topically delivered drug moiety makes them an appropriate drug delivery tool for ocular therapeutics. Polymeric nanoparticles also protect the encapsulated drug from the enzymes present in tears, allowing their prolonged and controlled release [111,112,113]. These nanoparticles also show the capacity to deliver the drug into deeper tissues [114]. It forms a depot, from which the drug is slowly delivered to the affected region over a period of time, reducing the frequency of administration, and facilitating drug targeting [115]. These particles generally range from 100–500 nm in size [109]. Morphologically, polymeric nanoparticles are classified into two distinct categories, namely, nanospheres if the polymeric core is based on a continuous network, and nanocapsules if the core is liquid or semisolid in which the drug is solubilized surrounded by a polymeric shell that controls the release of the drug. Figure 5 shows a schematic representation of both types.

In comparison with classical ophthalmic formulations, polymeric nanoparticles improve bioavailability without blurring the vision [116]. Owing to their appropriate particle size, polymeric nanoparticles are reported to show low or no irritation, a desirable feature of ophthalmic formulations (particles having size greater than 10 μm may result in patient discomfort) [117,118]. The nanoparticles could be internalized by the corneal cells and form a reservoir from which the drug could be released over time [119]. In addition to the particle size, lipophilicity, and surface charge, ocular bioavailability of polymeric nanoparticles relies mostly on the bioadhesion competence of the polymers. Without this characteristic feature, nanoparticles are eliminated from the eyes as quickly as an aqueous phase [120].

Among polymeric nanoparticles, nanospheres are composed of a vesicular architecture, with a polymer surrounding a liquid (hydrophilic or lipophilic) core (Figure 4, right). In such nanostructures, the drug moiety can either be dissolved inside or entrapped or adsorbed on the surface of particles [121].

Semi-synthetic and synthetic polymers like poly lactic acid (PLA), poly glycolic acid (PGA), and poly lactic acid-*co*-glycolic acid (PLGA) are widely employed as components of delivery vehicles for ocular administration [19,53,57,78,122,123,124]. However, nanotechnologists are mostly interested in hydrophilic polymers of natural sources because of their accomplished characteristics of affordability, biocompatibility, little or no toxicity, easy availability, and non-irritant profile. In addition to mucoadhesion, hydrophilic polymers swell and form a gel-like layer on the nanoparticle surface, which reduces fluid entry into the polymer core, resulting in sustained drug release [125]. Numerous natural polymers, such as guar gum, alginate, and chitosan, were explored as ocular nano delivery agents [126,127,128]. Chitosan is another natural polymer which is cationic in nature, biodegradable, strongly bioadhesive, and of low toxicity, and it contributes to increasing drug absorption [129]. Nanoparticle fabrications with some of these hydrophilic polymers, however, require the use of cross-linking agents, such as glutaraldehyde, which may pose toxicological risks when applied to living cells [130].

The latest scientific research advocates polymeric nanoparticles for ocular delivery of numerous drug moieties (Table 3). There is, however, not yet any scientific literature reporting the loading of ergot alkaloids into polymeric nanoparticles, while their merits nevertheless promulgate them as a propitious alternative for the topical management of glaucoma.

Polymeric nanoparticles dispersed in a thermosensitive gel were reported to be superior to commercial eye drops [46]. Olopatadine hydrochloride, a histamine H_1_ receptor antagonist with an effect on human conjunctival mast cells used in eye allergies, was loaded into in situ gel polymeric formulations to reduce the need for frequent application. Such in situ formulations generally allow gelation at corneal temperature [51], thus avoiding the burst release of the loaded drug in the initial hours of administration. As a result, higher drug concentration in deeper regions of eye (aqueous humor) may take place [52,131]. This system was sequentially employed to increase the bioavailability of hydrophobic drugs, particularly corticosteroids for ocular disorders [115]. The presence of a gel on the ocular surface may, however, cause loss of visual field; the rheological behavior must, therefore, be critically evaluated in order to improve ocular residence time and reduce blink resistance and blurred vision. It was reported that a viscosity of ca. 12–15 mPa·s is ideal for ophthalmic gels [47].

Recently, core–shell lipid–polymeric nanoparticles, combining the features of both polymeric and lipid nanoparticles, were also the focus of intensive research. Lipid–polymeric nanoparticles are composed of a lipid core and phospholipid shell [132]. The polymer core contributes to a stable architecture, with internal entrapment of hydrophobic/hydrophilic drug, and the phospholipid outer shell provides an improved drug embedding feature and biocompatibility. As a result, these nanoparticles hold great promise for topical drug administration onto the eye.

The surface charge of nanoparticles has an important role in adhesion and penetration of these nanostructures through the skin and mucus membranes. The corneal epithelium is negatively charged (under normal physiological conditions); thus, positively charged polymeric nanoparticles easily adhere to it. This helps in enhancing the residence time and increasing the drug concentration in the eye. Several polymers with different polarity, solubility, biodegradability, biocompatibility, swelling, and electrical charge can be employed for the crafting of polymeric nanoparticles [111].

The literature documents the growing interest of research community in cationic polymeric nanoparticles for the ocular delivery of a variety of compounds. One of the attractive features of cationic nanoparticles is the potential to modulate their surface for tuning the morphological and physicochemical attributes, in terms of surface charge, size, and shape, which affect their therapeutic utility to a large extent. Surface modification employing cationic polymers was proposed as a useful strategy to enhance nanoparticle stability and their interaction with biological membranes. For the design of this type of nanoparticle, the selection of the appropriate polymer, preferably biodegradable and biocompatible, assumes great significance [133].

## 4. Conclusions

The increased IOP in glaucoma can lead to irreversible damage of the ocular nerves. Natural ergot alkaloids were found to reduce the IOP mainly by a significant reduction in the aqueous humor inflow. Based on their remarkable intraocular pressure-lowering effect, these compounds could have a role in anti-glaucoma therapy. Despite the little literature available on ergot alkaloid delivery research, the encapsulation of these natural compounds in nanoparticles could contribute to a safer and more efficient alternative for the treatment of glaucoma via the ocular route. Among lipid nanoparticles, SLNs and NLCs are solid at room and body temperature, which contributes to modulating the release profile of the loaded drug. Furthermore, these nanoparticles demonstrated efficiency to enhance the bioavailability of many drugs, and the physiological composition of the raw materials offers reduced toxicity and high ocular tolerance. Polymeric nanoparticles also offer the opportunity to modulate the release profile of the loaded drugs. The use of cationic nanoparticles (lipid, polymeric) was exploited to improve the residence time of the particles in the eye, attributed to the electrostatic interaction with the anionic ocular mucosa. Considering the present status of natural ergot alkaloid and nanoparticle research, further clinical aspects would need to be assessed in the field of ocular administration, a very promising research field definitely worth of being explored.

## Figures and Tables

**Table 1:** Composition, mean particle size, polydispersity index and zeta potential of lipid nanoparticles (SLNs and NLCs) loading ophthalmic drugs for ocular administration. (NA, not available).

Encapsulated Drug	Lipid System Composition	Average Particle Size (nm)	Polydispersity Index	Zeta Potential (mV)	References
Brimonidine	NLC: Glyceryl monostearate, castor oil, and Poloxamer^®^ 188	152.0	0.230	−44.20	[79]
Curcumin	NLC: Compritol^®^ ATO 888, Gelucire^®^ 50/13, olive oil, and Poloxamer^®^ 188	66.8	0.170	NA	[80]
Etoposide	SLN: Gelucire 44/14, Compritol^®^ ATO 888, and Tween^®^ 80	239.4	0.261	NA	[14]
Flurbiprofen	NLC: stearic acid, castor oil, and Tween^®^ 80	288.0	0.245	−29.00	[53]
Isoniazid	SLN: Compritol^®^ ATO 888, stearic acid, Tween^®^ 80, and soy lecithin	149.2	0.150	−0.35	[81]
Natamycin	SLN: Precirol^®^ ATO 5 and Pluronic^®^ F68	84.0	0.224	26.70	[82]
Propranolol	NLC: Compritol^®^ ATO 888, oleic acid, Tween^®^ 80, Span^®^ 80, and Transcutol P	385.0 to 880.0	0.220 to 0.560	NA	[83]
Quercetin	NLC: Compritol^®^ ATO 888, medium-chaintriglyceride, Cremophor EL, and soy lecithin	75.5	0.180	NA	[84]
Sunitinib	SLN: Stearic acid, sodium taurodeoxycholate, and phosphatidylcholine	140.0	0.200	NA	[85]
Triamcinolone acetonide	SLN: Glyceryl monostearate, Compritol^®^ ATO 888, Tween^®^ 80, and Pluronic^®^ F68	200.0 to 350.0	0.300 to 0.450	−52.31 to −64.35	[86]
Voriconazole	SLN: Witepsol^®^ W35, Compritol^®^ ATO 888, stearic acid, Tween^®^ 80, and l-α-phosphatidylcholine	182.0	0.269	NA	[87]

**Table 2:** Composition, mean particle size, polydispersity index, and zeta potential of cationic lipid nanoparticles loading ophthalmic drugs for ocular administration. (NA, not available).

Encapsulated Drug	Cationic Lipid Nanoparticles	Average Particle Size (nm)	Polydispersity Index	Zeta Potential (mV)	References
Epigallocatechin gallate	Solid lipid nanoparticles (SLN): Ascorbic acid, Poloxamer 188, Softisan^®^ S75, CTAB (cetyl trimethylammonium bromide), DDAB (dimethyl dioctadecyl ammonium bromide)	149.0 and 143.0	0.240 and 0.160	20.80 and 25.70	[67]
Palmatine	Lipid emulsion: Egg lecithin, oleic acid, α- tocopherol, soybean oil, DOTAP (1,2-dioleoyl-3-trimethylammonium-propane)	192.4	0.281	45.00	[88]
NA	Nanoemulsion: Stearylamine, sesame oil, soybean oil, castor oil, Cremophor^®^EL, Tween 60, Tween 80, glycerol, Pluronic^®^ F68	81.0 to 96.0	0.110 to 0.251	−2.00 to 27.00	[104]
Curcumin	Nanostructured lipid carriers (NLC): Glycerinmonostearate, Tween 80, stearic acid, tristearin, Comritol^®^ 888 ATO, Miglyol^®^ 812	158.1	0.290	36.50	[93]
Besifloxacin hydrochloride	Nanostructured lipid carriers (NLC): Compritol 888 ATO, Gelucire 50/13, Labrafac PG, CTAB (cetyl trimethylammonium bromide)	173.6	0.188	16.60	[105]
NA	Solid lipid nanoparticles (SLN): Glycerol, Softisan S100, Lipoid S75, soybean phosphatidylcholine	134.0 and 135.0	0.179 and 0.196	28.20	[100]
Ciprofloxacin hydrochloride	Solid lipid nanoparticles (SLN): Tween 80, Softisan 100, TEA (trimethylamine), DDAB (dimethyl dioctadecyl ammonium bromide)	270.0 to 350.0	0.250 to 0.340	−42.00 to 51.00	[106]
NA	Solid lipid nanoparticles (SLN): Glycerol, Softisan S100, Lipoid S75, soybean phosphatidylcholine	164.5 to 268.5	0.155 to 0.192	−1.00 to −2.00	[66]
Puearin and Scutellarin	Solid lipid nanoparticles (SLN): F-127, Gelucire^®^ 44/14, Tween 80, cholesterol, quaternized carboxymethyl chitosan, lecithin	181.0	0.224	23.80	[95]
Epigallocatechin gallate	Solid lipid nanoparticles (SLN): Glycerol, Softisan S100, Lipoid S75, ascorbic acid, Poloxamer 188, DDAB (dimethyl dioctadecyl ammonium bromide)	143.7	0.160	25.70	[107]
NA	Solid lipid nanoparticles (SLN): Precirol ATO 5, Brij 76, CTAB (cetyl trimethylammonium bromide), DDAB (dimethyl dioctadecyl ammonium bromide)	185.0 to 244.0	0.350	16.00 to 55.00	[108]
Triamcinolone acetonide	Nanostructured lipid carriers (NLC): Transcutol^®^ P, Capmul MCM C10 (Glycerylmonocaprate), Captex 200 P, lecithin, Tween 80, stearylamine, ethanol	199.0	0.326	35.80	[91]

**Table 3:** Composition, mean particle size, polydispersity index, and zeta potential of polymeric nanoparticles loading ophthalmic drugs for ocular administration.

Encapsulated Drug	Polymeric Nanoparticle Composition	Average Particle Size (nm)	Polydispersity Index	Zeta Potential (mV)	References
Acetazolamide	Nanocapsule suspensions: Ethyl cellulose, Eudragit^®^ RS100, Tween 80, Span 60, medium-chain triglycerides	106.0 to 229.0	0.076 to 0.195	−16.20 to 17.30	[109]
Dirithromycin	Nanoparticles: Kollidon^®^ SR, methanol	329.6 and 522.2	0.425 and 0.539	−19.50 and −25.50	[134]
Fluorometholone	Nanoparticles: Polylactic-*co*-glycolic acid (PLGA), Poloxamer 188, acetone	150.8	0.082	−27.90	[135]
Gatifloxacin	Nanoparticles: Eudragit^®^ RL and RS, acetone, Tween- 80	68.0 and 410.0	0.408 and 0.280	24.45 and 33.30	[118]
Moxifloxacin	Hyaluronic-acid-modified lipid–polymer hybrid nanoparticles (HA-LCS-NPs): Carbodiimide hydrochloride, *N*-hydroxysuccinimide, DPPE (dipalmitoyl phosphatidylethanolamine), hyaluronic acid	214.0	0.394	−16.59	[110]
Melatonin	Aqueous core nanocapsules: Polyactic acid, Tween 80, Span 80, Brij^®^ 98, caprylic/capric triglyceride, CTAB (cetyl trimethyl ammonium bromide), DDAB (dimethyl dioctadecyl ammonium bromide)	193.0 to 218.0	0.16 to 0.22	−36.1 to 31.4	[133]
Brinzolamide	Nanoparticles: Poly(lactic-*co*-glycolic) acid (PLGA), acetone, polyvinyl alcohol, soybean phosphatidylcholine, cholesterol	151.0	NA	−7.53	[136]
Delonix galacto mannan	Nanoparticles: Delonix polymer, sodium hydroxide, Tween 80, Span 80, ethanol	215.0 to 360.0	0.140 to 0.225	−68.80 to −31.80	[130]
Fluocinolone acetonide	Nanoparticles: PLGA, poloxamer, acetonitrile	34.0 to 178.0	0.16 to 0.52	−1.43 to 5.25	[137]
Flurbiprofen	Nanoparticles: Poly ε-caprolactone, Poloxamer 188, acetone	170.6 and 192.5	0.087 and 0.139	−15.50 and −12.00	[115]
Dexamethasone	Nanoparticles: Ethyl cellulose, Eudragit^®^ RS, ethyl acetate, polyvinyl alcohol	64.0 to 172.0	0.058 to 0.218	−36.00 to 44.00	[111]
5-Fluorouracil	Nanoparticles: Sulfobutyl chitosan, polymer solution, 1,4-butane sulfone, phosphate buffer pH 7.4	294.3 to 390.6	0.2 to 0.4	−3.50 to +9.50	[119]
Vancomycin	Nanoparticles: Poly lactic-*co*-glycolic acid (PLGA), polycaprolactone, Span 80, acetonitrile, methylene dichloride, liquid paraffin	155.0 to 8444.0	0.222 to 0.893	−43.15 to 51.55	[120]
Pilocarpine hydrochloride	Nanoparticles: Eudragit^®^ RS100, Gelucire^®^ 44/14, Tween 80, benzalkonium chloride, octadecylamine	73.0 to 3179.0	0.29 to 1	−2.97 to 85.00	[116]
Moxifloxacin hydrochloride	Nanoparticles: Eudragit^®^ RS100, methanol	247.1 to 392.4	0.23 to 0.68	53.90 to 80.40	[138]
Timolol maleate	Nanoparticles: Flax seed gum, acetic acid	254.5	0.345	−20.30	[139]

## References

[1] Jakubczyk D., Dussart F. (2020). Selected Fungal Natural Products with Antimicrobial Properties. Molecules.

[2] Robinson S.L., Panaccione D.G. (2015). Diversification of ergot alkaloids in natural and modified fungi. Toxins.

[3] Crews C. (2015). Analysis of Ergot Alkaloids. Toxins.

[4] Schiff P.L. (2006). Ergot and its alkaloids. Am. J. Pharm. Educ..

[5] Agriopoulou S., Stamatelopoulou E., Varzakas T. (2020). Advances in Occurrence, Importance, and Mycotoxin Control Strategies: Prevention and Detoxification in Foods. Foods.

[6] Carballo D., Tolosa J., Ferrer E., Berrada H. (2019). Dietary exposure assessment to mycotoxins through total diet studies. A review. Food Chem. Toxicol..

[7] Scott P. (2009). Ergot alkaloids: Extent of human and animal exposure. World Mycotoxin J..

[8] Mikušová P., Ritieni A., Santini A., Juhasová G., Šrobárová A. (2010). Contamination by moulds of grape berries in Slovakia. Food Addit. Contam. Part A.

[9] Mikušová P., Šrobárová A., Sulyok M., Santini A. (2013). Fusarium fungi and associated metabolites presence on grapes from Slovakia. Mycotoxin Res..

[10] Santini A., Ferracane R., Meca G., Ritieni A. (2009). Overview of analytical methods for beauvericin and fusaproliferin in food matrices. Anal. Bioanal. Chem..

[11] Rodríguez-Carrasco Y., Gaspari A., Graziani G., Santini A., Ritieni A. (2018). Fast analysis of polyphenols and alkaloids in cocoa-based products by ultra-high performance liquid chromatography and Orbitrap high resolution mass spectrometry (UHPLC-Q-Orbitrap-MS/MS). Food Res. Int..

[12] Ritieni A., Santini A., Mussap M., Ferracane R., Bosco P., Gazzolo D., Galvano F. (2010). Simultaneous determination of mycotoxins in biological fluids by LC-MS/MS. Front. Biosci. (Elite Ed.).

[13] Amirkia V., Heinrich M. (2014). Alkaloids as drug leads—A predictive structural and biodiversity-based analysis. Phytochem. Lett..

[14] Ahmad S., Garg M., Tamboli E.T., Abdin M.Z., Ansari S.H. (2013). In vitro production of alkaloids: Factors, approaches, challenges and prospects. Pharmacogn. Rev..

[15] Adamski Z., Blythe L.L., Milella L., Bufo S.A. (2020). Biological Activities of Alkaloids: From Toxicology to Pharmacology. Toxins.

[16] Klotz J.L. (2015). Activities and Effects of Ergot Alkaloids on Livestock Physiology and Production. Toxins.

[17] Mulac D., Hüwel S., Galla H.-J., Humpf H.-U. (2012). Permeability of ergot alkaloids across the blood-brain barrier in vitro and influence on the barrier integrity. Mol. Nutr. Food Res..

[18] Mulac D., Humpf H.U. (2011). Cytotoxicity and accumulation of ergot alkaloids in human primary cells. Toxicology.

[19] Sanchez-Lopez E., Egea M.A., Davis B.M., Guo L., Espina M., Silva A.M., Calpena A.C., Souto E.M.B., Ravindran N., Ettcheto M. (2018). Memantine-Loaded PEGylated Biodegradable Nanoparticles for the Treatment of Glaucoma. Small.

[20] Sánchez-López E., Lopez-Machado A.L., Bonilla V.L., Pizarro P.G., Silva A.M., Souto E.B. (2020). Lipid nanoparticles as carriers for the treatment of neurodegeneration associated with Alzheimer’s disease and glaucoma: Present and future challenges. Curr. Pharm. Des..

[21] Hertzog L.H., Albrecht K.G., LaBree L., Lee P.P. (1996). Glaucoma care and conformance with preferred practice patterns. Examination of the private, community-based ophthalmologist. Ophthalmology.

[22] Singh R.B., Ichhpujani P., Thakur S., Jindal S. (2020). Promising therapeutic drug delivery systems for glaucoma: A comprehensive review. Ther. Adv. Ophthalmol..

[23] McAlinden C. (2014). Selective laser trabeculoplasty (SLT) vs. other treatment modalities for glaucoma: Systematic review. Eye (Lond. Engl.).

[24] Weinreb R.N., Leung C.K.S., Crowston J.G., Medeiros F.A., Friedman D.S., Wiggs J.L., Martin K.R. (2016). Primary open-angle glaucoma. Nat. Rev. Dis. Primers.

[25] Thiel R. (1926). Experimental and clinical investigations on the influence of ergotamine (Gynergen) on the intraocular pressure in glaucoma. Klin. Mon. Augenh..

[26] Kappert A., Hadron W. (1950). Experimental and therapeutic investigations with certain new hydrogenated ergot alkaloids in peripheral vascular disorders. Angiology.

[27] Chen J.-J., Han M.-Y., Gong T., Yang J.-L., Zhu P. (2017). Recent progress in ergot alkaloid research. RSC Adv..

[28] Berde B. (1978). Pharmacology of ergot alkaloids in clinical use. Med. J. Aust..

[29] Mukherjee J., Menge M. (2000). Progress and Prospects of Ergot Alkaloid Research. New Products and New Areas of Bioprocess Engineering.

[30] Diotallevi M., Auricchio G. (1964). The effect of topically used hydergine on ocular tension. Ophthalmologica.

[31] Al-Sereiti M.R., Quik R.F., Turner P. (1989). The effect of a single oral dose of pergolide on intraocular pressure and pupil diameter. Br. J. Clin. Pharmacol..

[32] Elibol O., Güler C., Yüksel N. (1992). The effects of dopamine, haloperidol and bromocriptine on intraocular pressure. Int. Ophthalmol..

[33] Potter D.E., Ogidigben M.J., Chu T.C. (1998). Lisuride acts at multiple sites to induce ocular hypotension and mydriasis. Pharmacology.

[34] Potter D.E., Shumate D.J. (1987). Cianergoline Lowers Intraocular Pressure in Rabbits and Monkeys and Inhibits Contraction of the Cat Nictitans by Suppressing Sympathetic Neuronal Function. J. Ocul. Pharmacol. Ther..

[35] Cavanak T. (1987). Occular Formulation Comprising Bromocriptine. U.S. Patent.

[36] Gerhards N., Neubauer L., Tudzynski P., Li S.-M. (2014). Biosynthetic Pathways of Ergot Alkaloids. Toxins.

[37] Al-Sereiti M.R., Coakes R.L., O’Sullivan D.P., Turner P. (1989). A comparison of the ocular hypotensive effect of 0.025% bromocriptine and 0.25% timolol eye drops in normal human volunteers. Br. J. Clin. Pharmacol..

[38] Mekki Q.A., Warrington S.J., Turner P. (1984). Bromocriptine eyedrops lower intraocular pressure without affecting prolactin levels. Lancet (Lond. Engl.).

[39] Puras G., Santafé J., Segarra J., Garrido M., Melena J. (2002). The effect of topical natural ergot alkaloids on the intraocular pressure and aqueous humor dynamics in rabbits with alpha-chymotrypsin-induced ocular hypertension. Graefe’s Arch. Clin. Exp. Ophthalmol..

[40] Puras G., Santafé J., Segarra J., Garrido M., Melena J. (2007). A comparative study of topical natural ergot alkaloids on the intraocular pressure and aqueous humor dynamics in oclular normotensive and alpha-chymotrypsin-induced ocular hypertensive rabbits. Graefe’s Arch. Clin. Exp. Ophthalmol..

[41] Puras G., Cassiano N.M. (2011). Intraocular pressure lowering effect of natural ergot alkaloids and their future applications in ocular pharmacology. Alkaloids: Properties, Applications and Pharmacological Effects.

[42] Santafé Oroz J., Segarra Domenech J., Garrido García M., Pablo Martínez V. (1991). Effects of topical dihydroergocristine on intraocular pressure, aqueous humor dynamics and pupil diameter in conscious rabbits. A comparative study with timolol and pilocarpine. Methods Find. Exp. Clin. Pharmacol..

[43] Rowell P.P., Larson B.T. (1999). Ergocryptine and other ergot alkaloids stimulate the release of [3H]dopamine from rat striatal synaptosomes2. J. Anim. Sci..

[44] Abul Kalam M., Sultana Y., Ali A., Aqil M., Mishra A.K., Chuttani K., Aljuffali I.A., Alshamsan A. (2013). Part II: Enhancement of transcorneal delivery of gatifloxacin by solid lipid nanoparticles in comparison to commercial aqueous eye drops. J. Biomed. Mater. Res. Part A.

[45] Pita-Thomas D.W., Goldberg J.L. (2013). Nanotechnology and glaucoma: Little particles for a big disease. Curr. Opin. Ophthalmol..

[46] Güven U.M., Berkman M.S., Enel B., Yazan Y. (2019). Development and in vitro/in vivo evaluation of thermo-sensitive in situ gelling systems for ocular allergy. Braz. J. Pharm. Sci..

[47] McKenzie B., Kay G. (2015). Eye gels for ophthalmic delivery. Expert Rev. Ophthalmol..

[48] Sanchez-Lopez E., Espina M., Doktorovova S., Souto E.B., Garcia M.L. (2017). Lipid nanoparticles (SLN, NLC): Overcoming the anatomical and physiological barriers of the eye—Part I-Barriers and determining factors in ocular delivery. Eur. J. Pharm. Biopharm..

[49] Joseph M., Trinh H.M., Cholkar K., Pal D., Mitra A.K. (2016). Recent perspectives on the delivery of biologics to back of the eye. Expert Opin. Drug Deliv..

[50] Souto E.B., Souto S.B., Severino P., Dias-Ferreira J., Naveros B.C., Durazzo A., Lucarini M., Atanasov A.G., El Mamouni S., Santini A. (2020). Croton argyrophyllus Kunth essential oil - loaded SLN: Optimization and evaluation of antioxidant and antitumoral activities. Molecules.

[51] Ghate D., Edelhauser H.F. (2006). Ocular drug delivery. Expert Opin. Drug Deliv..

[52] Sánchez-López E., Esteruelas G., Ortiz A., Espina M., Prat J., Muñoz M., Cano A., Calpena A.C., Ettcheto M., Camins A. (2020). Dexibuprofen Biodegradable Nanoparticles: One Step Closer towards a Better Ocular Interaction Study. Nanomaterials.

[53] Gonzalez-Mira E., Egea M.A., Garcia M.L., Souto E.B. (2010). Design and ocular tolerance of flurbiprofen loaded ultrasound-engineered NLC. Colloids Surf B Biointerfaces.

[54] Souto E.B., Dias-Ferreira J., Lopez-Machado A., Ettcheto M., Cano A., Camins Espuny A., Espina M., Garcia M.L., Sanchez-Lopez E. (2019). Advanced Formulation Approaches for Ocular Drug Delivery: State-of-the-Art and Recent Patents. Pharmaceutics.

[55] Sanchez-Lopez E., Espina M., Doktorovova S., Souto E.B., Garcia M.L. (2017). Lipid nanoparticles (SLN, NLC): Overcoming the anatomical and physiological barriers of the eye—Part II—Ocular drug-loaded lipid nanoparticles. Eur. J. Pharm. Biopharm..

[56] Fangueiro J.F., Veiga F., Silva A.M., Souto E.B. (2016). Ocular Drug Delivery—New Strategies for Targeting Anterior and Posterior Segments of the Eye. Curr. Pharm. Des..

[57] Gonzalez-Mira E., Egea M.A., Souto E.B., Calpena A.C., Garcia M.L. (2011). Optimizing flurbiprofen-loaded NLC by central composite factorial design for ocular delivery. Nanotechnology.

[58] Vieira R., Severino P., Nalone L.A., Souto S.B., Silva A.M., Lucarini M., Durazzo A., Santini A., Souto E.B. (2020). Sucupira Oil-Loaded Nanostructured Lipid Carriers (NLC): Lipid Screening, Factorial Design, Release Profile, and Cytotoxicity. Molecules.

[59] Souto E.B., Souto S.B., Zielinska A., Durazzo A., Lucarini M., Santini A., Horbańczuk O.K., Atanasov A.G., Marques C., Andrade L.N. (2020). Perillaldehyde 1,2-epoxide loaded SLN-tailored mAb: Production, physicochemical characterization and in vitro cytotoxicity profile in MCF-7 cell lines. Pharmaceutics.

[60] Zielinska A., Ferreira N.R., Durazzo A., Lucarini M., Cicero N., Mamouni S.E., Silva A.M., Nowak I., Santini A., Souto E.B. (2019). Development and Optimization of Alpha-Pinene-Loaded Solid Lipid Nanoparticles (SLN) Using Experimental Factorial Design and Dispersion Analysis. Molecules.

[61] Rigon R.B., Fachinetti N., Severino P., Durazzo A., Lucarini M., Atanasov A.G., El Mamouni S., Chorilli M., Santini A., Souto E.B. (2019). Quantification of Trans-Resveratrol-Loaded Solid Lipid Nanoparticles by a Validated Reverse-Phase HPLC Photodiode Array. Appl. Sci..

[62] Campos J.R., Severino P., Santini A., Silva A.M., Shegokar R., Souto S.B., Souto E.B., Shegokar R. (2020). Chapter 1—Solid lipid nanoparticles (SLN): Prediction of toxicity, metabolism, fate and physicochemical properties. Nanopharmaceuticals.

[63] Wadetwar R.N., Agrawal A.R., Kanojiya P.S. (2020). In situ gel containing Bimatoprost solid lipid nanoparticles for ocular delivery: In-vitro and ex-vivo evaluation. J. Drug Deliv. Sci. Technol..

[64] Doktorovova S., Santos D.L., Costa I., Andreani T., Souto E.B., Silva A.M. (2014). Cationic solid lipid nanoparticles interfere with the activity of antioxidant enzymes in hepatocellular carcinoma cells. Int. J. Pharm..

[65] Doktorovova S., Shegokar R., Rakovsky E., Gonzalez-Mira E., Lopes C.M., Silva A.M., Martins-Lopes P., Muller R.H., Souto E.B. (2011). Cationic solid lipid nanoparticles (cSLN): Structure, stability and DNA binding capacity correlation studies. Int. J. Pharm..

[66] Fangueiro J.F., Andreani T., Egea M.A., Garcia M.L., Souto S.B., Silva A.M., Souto E.B. (2014). Design of cationic lipid nanoparticles for ocular delivery: Development, characterization and cytotoxicity. Int. J. Pharm..

[67] Fangueiro J.F., Calpena A.C., Clares B., Andreani T., Egea M.A., Veiga F.J., Garcia M.L., Silva A.M., Souto E.B. (2016). Biopharmaceutical evaluation of epigallocatechin gallate-loaded cationic lipid nanoparticles (EGCG-LNs): In vivo, in vitro and ex vivo studies. Int. J. Pharm..

[68] Araujo J., Garcia M.L., Mallandrich M., Souto E.B., Calpena A.C. (2012). Release profile and transscleral permeation of triamcinolone acetonide loaded nanostructured lipid carriers (TA-NLC): In vitro and ex vivo studies. Nanomedicine.

[69] Araujo J., Nikolic S., Egea M.A., Souto E.B., Garcia M.L. (2011). Nanostructured lipid carriers for triamcinolone acetonide delivery to the posterior segment of the eye. Colloids Surf. B Biointerfaces.

[70] Doktorovova S., Araujo J., Garcia M.L., Rakovsky E., Souto E.B. (2010). Formulating fluticasone propionate in novel PEG-containing nanostructured lipid carriers (PEG-NLC). Colloids Surf. B Biointerfaces.

[71] Araujo J., Gonzalez-Mira E., Egea M.A., Garcia M.L., Souto E.B. (2010). Optimization and physicochemical characterization of a triamcinolone acetonide-loaded NLC for ocular antiangiogenic applications. Int. J. Pharm..

[72] Araujo J., Gonzalez E., Egea M.A., Garcia M.L., Souto E.B. (2009). Nanomedicines for ocular NSAIDs: Safety on drug delivery. Nanomedicine.

[73] Souto E.B., Almeida A.J., Müller R.H. (2007). Lipid Nanoparticles (SLN^®^, NLC^®^) for Cutaneous Drug Delivery: Structure, Protection and Skin Effects. J. Biomed. Nanotechnol..

[74] Souto E.B., Baldim I., Oliveira W.P., Rao R., Yadav N., Gama F.M., Mahant S. (2020). SLN and NLC for topical, dermal and transdermal drug delivery. Expert Opin. Drug Deliv..

[75] Zielińska A., Ferreira N.R., Feliczak-Guzik A., Nowak I., Souto E.B. (2020). Loading, release profile and accelerated stability assessment of monoterpenes-loaded Solid Lipid Nanoparticles (SLN). Pharm. Dev. Technol..

[76] Souto E.B., Doktorovova S., Gonzalez-Mira E., Egea M.A., Garcia M.L. (2010). Feasibility of lipid nanoparticles for ocular delivery of anti-inflammatory drugs. Curr. Eye Res..

[77] Fangueiro J.F., Andreani T., Fernandes L., Garcia M.L., Egea M.A., Silva A.M., Souto E.B. (2014). Physicochemical characterization of epigallocatechin gallate lipid nanoparticles (EGCG-LNs) for ocular instillation. Colloids Surf. B Biointerfaces.

[78] Doktorovova S., Kovacevic A.B., Garcia M.L., Souto E.B. (2016). Preclinical safety of solid lipid nanoparticles and nanostructured lipid carriers: Current evidence from in vitro and in vivo evaluation. Eur. J. Pharm. Biopharm..

[79] El-Salamouni N.S., Farid R.M., El-Kamel A.H., El-Gamal S.S. (2018). Nanostructured lipid carriers for intraocular brimonidine localisation: Development, in-vitro and in-vivo evaluation. J. Microencapsul..

[80] Lakhani P., Patil A., Taskar P., Ashour E., Majumdar S. (2018). Curcumin-loaded Nanostructured Lipid Carriers for ocular drug delivery: Design optimization and characterization. J. Drug Deliv. Sci. Technol..

[81] Singh M., Guzman-Aranguez A., Hussain A., Srinivas C.S., Kaur I.P. (2019). Solid lipid nanoparticles for ocular delivery of isoniazid: Evaluation, proof of concept and in vivo safety & kinetics. Nanomedicine (Lond. Engl.).

[82] Khames A., Khaleel M.A., El-Badawy M.F., El-Nezhawy A.O.H. (2019). Natamycin solid lipid nanoparticles—Sustained ocular delivery system of higher corneal penetration against deep fungal keratitis: Preparation and optimization. Int. J. Nanomed..

[83] Sharif Makhmal Zadeh B., Niro H., Rahim F., Esfahani G. (2018). Ocular Delivery System for Propranolol Hydrochloride Based on Nanostructured Lipid Carrier. Sci. Pharm..

[84] Yu Y., Feng R., Yu S., Li J., Wang Y., Song Y., Yang X., Pan W., Li S. (2018). Nanostructured lipid carrier-based pH and temperature dual-responsive hydrogel composed of carboxymethyl chitosan and poloxamer for drug delivery. Int. J. Biol. Macromol..

[85] Freitas L.G.A.D., Isaac D.L.C., Lima E.M., Souza L.G., Abud M.A., Reis R.G.D., Tannure W.T., Ávila M.P.D. (2018). Retinal changes in rabbit after intravitreal injection of sunitinib encapsulated into solid lipid nanoparticles and polymeric nanocapsules. Arq. Bras. Oftalmol..

[86] Tatke A., Dudhipala N., Janga K.Y., Balguri S.P., Avula B., Jablonski M.M., Majumdar S. (2018). In Situ Gel of Triamcinolone Acetonide-Loaded Solid Lipid Nanoparticles for Improved Topical Ocular Delivery: Tear Kinetics and Ocular Disposition Studies. Nanomaterials.

[87] Füredi P., Pápay Z.E., Kovács K., Kiss B.D., Ludányi K., Antal I., Klebovich I. (2017). Development and characterization of the voriconazole loaded lipid-based nanoparticles. J. Pharm. Biomed. Anal..

[88] Yin J., Xiang C., Lu G. (2016). Cationic lipid emulsions as potential bioadhesive carriers for ophthalmic delivery of palmatine. J. Microencapsul..

[89] Chhonker Y.S., Prasad Y.D., Chandasana H., Vishvkarma A., Mitra K., Shukla P.K., Bhatta R.S. (2015). Amphotericin-B entrapped lecithin/chitosan nanoparticles for prolonged ocular application. Int. J. Biol. Macromol..

[90] Li H., Liu W., Sorenson C.M., Sheibani N., Albert D.M., Senanayake T., Vinogradov S., Henkin J., Zhang H.F. (2017). Sustaining Intravitreal Residence with L-Arginine Peptide-Conjugated Nanocarriers. Investig. Ophthalmol. Vis. Sci..

[91] Nirbhavane P., Sharma G., Singh B., Begum G., Jones M.-C., Rauz S., Vincent R., Denniston A.K., Hill L.J., Katare O.P. (2020). Triamcinolone acetonide loaded-cationic nano-lipoidal formulation for uveitis: Evidences of improved biopharmaceutical performance and anti-inflammatory activity. Colloids Surf. B Biointerfaces.

[92] Klang S.H., Frucht-Pery J., Hoffman A., Benita S. (1994). Physicochemical Characterization and Acute Toxicity Evaluation of a Positively-charged Submicron Emulsion Vehicle. J. Pharm. Pharmacol..

[93] Liu R., Wang S., Sun L., Fang S., Wang J., Huang X., You Z., He X., Liu C. (2016). A novel cationic nanostructured lipid carrier for improvement of ocular bioavailability: Design, optimization, in vitro and in vivo evaluation. J. Drug Deliv. Sci. Technol..

[94] Avachat A.M., Parpani S.S. (2015). Formulation and development of bicontinuous nanostructured liquid crystalline particles of efavirenz. Colloids Surf. B Biointerfaces.

[95] Wang J., Zhao F., Liu R., Chen J., Zhang Q., Lao R., Wang Z., Jin X., Liu C. (2017). Novel cationic lipid nanoparticles as an ophthalmic delivery system for multicomponent drugs: Development, characterization, in vitro permeation, in vivo pharmacokinetic, and molecular dynamics studies. Int. J. Nanomed..

[96] Yaghmur A., Glatter O. (2009). Characterization and potential applications of nanostructured aqueous dispersions. Adv. Colloid Interface Sci..

[97] Doktorovova S., Souto E.B. (2009). Nanostructured lipid carrier-based hydrogel formulations for drug delivery: A comprehensive review. Expert Opin. Drug Deliv..

[98] Kalbáčová M., Verdánová M., Mravec F., Halasová T., Pekař M. (2014). Effect of CTAB and CTAB in the presence of hyaluronan on selected human cell types. Colloids Surf. A Physicochem. Eng. Asp..

[99] Nabi A., Tasneem S., Jesudason C.G., Lee V.S., Zain S.B.M. (2018). Study of interaction between cationic surfactant (CTAB) and paracetamol by electrical conductivity, tensiometric and spectroscopic methods. J. Mol. Liq..

[100] Silva A.M., Martins-Gomes C., Coutinho T.E., Fangueiro J.F., Sanchez-Lopez E., Pashirova T.N., Andreani T., Souto E.B. (2019). Soft cationic nanoparticles for drug delivery: Production and cytotoxicity of solid lipid nanoparticles (SLNs). Appl. Sci..

[101] Elansezhian R., Ramamoorthy B., Nair P.K. (2009). The influence of SDS and CTAB surfactants on the surface morphology and surface topography of electroless Ni–P deposits. J. Mater. Process. Technol..

[102] Oremusová J., Vitková Z., Vitko A., Tárník M., Miklovičová E., Ivánková O., Murgaš J., Krchňák D. (2019). Effect of Molecular Composition of Head Group and Temperature on Micellar Properties of Ionic Surfactants with C12 Alkyl Chain. Molecules.

[103] Liu Q., Liu S., Luo D., Peng B. (2019). Ultra-Low Interfacial Tension Foam System for Enhanced Oil Recovery. Appl. Sci..

[104] Dukovski B.J., Bračko A., Šare M., Pepić I., Lovrić J. (2019). In vitro evaluation of stearylamine cationic nanoemulsions for improved ocular drug delivery. Acta Pharm. (Zagrebcroatia).

[105] Baig M.S., Owida H., Njoroge W., Yang Y. (2020). Development and evaluation of cationic nanostructured lipid carriers for ophthalmic drug delivery of besifloxacin. J. Drug Deliv. Sci. Technol..

[106] Pignatello R., Leonardi A., Fuochi V., Petronio Petronio G., Greco A.S., Furneri P.M. (2018). A method for efficient loading of ciprofloxacin hydrochloride in cationic solid lipid nanoparticles: Formulation and microbiological evaluation. Nanomaterials.

[107] Silva A.M., Martins-Gomes C., Fangueiro J.F., Andreani T., Souto E.B. (2019). Comparison of antiproliferative effect of epigallocatechin gallate when loaded into cationic solid lipid nanoparticles against different cell lines. Pharm. Dev. Technol..

[108] Botto C., Mauro N., Amore E., Martorana E., Giammona G., Bondi M.L. (2017). Surfactant effect on the physicochemical characteristics of cationic solid lipid nanoparticles. Int. J. Pharm..

[109] Quinteros D.A., Ferreira L.M., Schaffazick S.R., Palma S.D., Allemandi D.A., Cruz L. (2016). Novel polymeric nanoparticles intended for ophthalmic administration of acetazolamide. J. Pharm. Sci..

[110] Liu D., Lian Y., Fang Q., Liu L., Zhang J., Li J. (2018). Hyaluronic-acid-modified lipid-polymer hybrid nanoparticles as an efficient ocular delivery platform for moxifloxacin hydrochloride. Int. J. Biol. Macromol..

[111] Balzus B., Sahle F.F., Hönzke S., Gerecke C., Schumacher F., Hedtrich S., Kleuser B., Bodmeier R. (2017). Formulation and ex vivo evaluation of polymeric nanoparticles for controlled delivery of corticosteroids to the skin and the corneal epithelium. Eur. J. Pharm. Biopharm..

[112] Gonzalez-Pizarro R., Silva-Abreu M., Calpena A.C., Egea M.A., Espina M., García M.L. (2018). Development of fluorometholone-loaded PLGA nanoparticles for treatment of inflammatory disorders of anterior and posterior segments of the eye. Int. J. Pharm..

[113] Trinh T.X., Choi J.S., Jeon H., Byun H.G., Yoon T.H., Kim J. (2018). Quasi-SMILES-Based Nano-Quantitative Structure-Activity Relationship Model to Predict the Cytotoxicity of Multiwalled Carbon Nanotubes to Human Lung Cells. Chem. Res. Toxicol..

[114] Tahara K., Karasawa K., Onodera R., Takeuchi H. (2017). Feasibility of drug delivery to the eye’s posterior segment by topical instillation of PLGA nanoparticles. Asian J. Pharm. Sci..

[115] Ramos G.Y., García M.L., Espina M.G., Parra A.C., Calpena A.C. (2016). Influence of freeze-drying and γ-irradiation in preclinical studies of flurbiprofen polymeric nanoparticles for ocular delivery using d-(+)-trehalose and polyethylene glycol. Int. J. Nanomed..

[116] Lütfi G., Müzeyyen D. (2013). Preparation and characterization of polymeric and lipid nanoparticles of pilocarpine HCl for ocular application. Pharm. Dev. Technol..

[117] Dillen K., Vandervoort J., Van den Mooter G., Ludwig A. (2006). Evaluation of ciprofloxacin-loaded Eudragit RS100 or RL100/PLGA nanoparticles. Int. J. Pharm..

[118] Duxfield L., Sultana R., Wang R., Englebretsen V., Deo S., Swift S., Rupenthal I., Al-Kassas R. (2016). Development of gatifloxacin-loaded cationic polymeric nanoparticles for ocular drug delivery. Pharm. Dev. Technol..

[119] Fabiano A., Piras A.M., Guazzelli L., Storti B., Bizzarri R., Zambito Y. (2019). Impact of Different Mucoadhesive Polymeric Nanoparticles Loaded in Thermosensitive Hydrogels on Transcorneal Administration of 5-Fluorouracil. Pharmaceutics.

[120] Yousry C., Elkheshen S.A., El-Laithy H.M., Essam T., Fahmy R.H. (2017). Studying the influence of formulation and process variables on Vancomycin-loaded polymeric nanoparticles as potential carrier for enhanced ophthalmic delivery. Eur. J. Pharm. Sci..

[121] Mora-Huertas C.E., Fessi H., Elaissari A. (2010). Polymer-based nanocapsules for drug delivery. Int. J. Pharm..

[122] Araujo J., Vega E., Lopes C., Egea M.A., Garcia M.L., Souto E.B. (2009). Effect of polymer viscosity on physicochemical properties and ocular tolerance of FB-loaded PLGA nanospheres. Colloids Surf. B Biointerfaces.

[123] Canadas C., Alvarado H., Calpena A.C., Silva A.M., Souto E.B., Garcia M.L., Abrego G. (2016). In vitro, ex vivo and in vivo characterization of PLGA nanoparticles loading pranoprofen for ocular administration. Int. J. Pharm..

[124] Sanchez-Lopez E., Egea M.A., Cano A., Espina M., Calpena A.C., Ettcheto M., Camins A., Souto E.B., Silva A.M., Garcia M.L. (2016). PEGylated PLGA nanospheres optimized by design of experiments for ocular administration of dexibuprofen-in vitro, ex vivo and in vivo characterization. Colloids Surf. B Biointerfaces.

[125] Croisfelt F.M., Tundisi L.L., Ataide J.A., Silveira E., Tambourgi E.B., Jozala A.F., Souto E.M.B., Mazzola P.G. (2019). Modified-release topical hydrogels: A ten-year review. J. Mater. Sci..

[126] Gratieri T., Gelfuso G.M., Rocha E.M., Sarmento V.H., de Freitas O., Lopez R.F.V. (2010). A poloxamer/chitosan in situ forming gel with prolonged retention time for ocular delivery. Eur. J. Pharm. Biopharm..

[127] Bhowmik M., Kumari P., Sarkar G., Bain M.K., Bhowmick B., Mollick M.M., Mondal D., Maity D., Rana D., Bhattacharjee D. (2013). Effect of xanthan gum and guar gum on in situ gelling ophthalmic drug delivery system based on poloxamer-407. Int. J. Biol. Macromol..

[128] Nagarwal R.C., Kant S., Singh P.N., Maiti P., Pandit J.K. (2009). Polymeric nanoparticulate system: A potential approach for ocular drug delivery. J. Control. Release.

[129] Teixeira M.D.C., Santini A., Souto E.B., Anton F., Alexandru G. (2017). Chapter 8—Delivery of Antimicrobials by Chitosan-Composed Therapeutic Nanostructures. Nanostructures for Antimicrobial Therapy.

[130] Ogunjimi A.T., Melo S.M.G., Vargas-Rechia C.G., Emery F.S., Lopez R.F.V. (2017). Hydrophilic polymeric nanoparticles prepared from Delonix galactomannan with low cytotoxicity for ocular drug delivery. Carbohydr. Polym..

[131] Nicoli S., Ferrari G., Quarta M., Macaluso C., Govoni P., Dallatana D., Santi P. (2009). Porcine sclera as a model of human sclera for in vitro transport experiments: Histology, SEM, and comparative permeability. Mol. Vis..

[132] Yang X.Z., Dou S., Wang Y.C., Long H.Y., Xiong M.H., Mao C.Q., Yao Y.D., Wang J. (2012). Single-step assembly of cationic lipid-polymer hybrid nanoparticles for systemic delivery of siRNA. ACS Nano.

[133] Carbone C., Manno D., Serra A., Musumeci T., Pepe V., Tisserand C., Puglisi G. (2016). Innovative hybrid vs. polymeric nanocapsules: The influence of the cationic lipid coating on the “4S”. Colloids Surf. B Biointerfaces.

[134] Basaran E. (2017). Ocular Application of Dirithromycin Incorporated Polymeric Nanoparticles: An in Vitro Evaluation/Diritromisin Yuklu Polimerik Nanopartikullerin Okuler Uygulanmasi: In Vitro Degerlendirme. Turk. J. Pharm. Sci..

[135] Gonzalez-Pizarro R., Carvajal-Vidal P., Bellowa L.H., Calpena A.C., Espina M., García M.L. (2019). In-situ forming gels containing fluorometholone-loaded polymeric nanoparticles for ocular inflammatory conditions. Colloids Surf. B Biointerfaces.

[136] Zhou Y., Fang A., Wang F., Li H., Jin Q., Huang L., Fu C., Zeng J., Jin Z., Song X. (2020). Core-shell lipid-polymer nanoparticles as a promising ocular drug delivery system to treat glaucoma. Chin. Chem. Lett..

[137] Salama A.H., Mahmoud A.A., Kamel R. (2016). A novel method for preparing surface-modified fluocinolone acetonide loaded PLGA nanoparticles for ocular use: In vitro and in vivo evaluations. AAPS PharmSciTech.

[138] Yurtdas-Kirimlioglu G., Sinan Ö., Büyükköroğlu G., Yazan Y. (2018). Formulation and in vitro evaluation of moxifloxacin hydrochloride-loaded polymeric nanoparticles for ocular application. Lat. Am. J. Pharm..

[139] Mittal N., Kaur G. (2019). Investigations on polymeric nanoparticles for ocular delivery. Adv. Polym. Technol..

